# Epidemiological survey of two morphotypes of *Demodex folliculorum* (Prostigmata: Demodicidade) in young people from southern Spain

**DOI:** 10.1016/j.parepi.2024.e00381

**Published:** 2024-09-28

**Authors:** F.J. Márquez, A.J. López-Montoya, S. Sánchez-Carrión, I. Dimov, M. de Rojas

**Affiliations:** a Department of Animal Biology, Vegetal Biology and Ecology, Universidad de Jaén, Spain; b Department of Statistics and Operational Research, Universidad de Jaén, Spain; c Department of Microbiology and Parasitology, Faculty of Pharmacy, University of Seville, Profesor García González 2, 41012 Seville, Spain; d Laboratory of Parasitology, Zoological Institute, Russian Academy of Science, Universitetskaya embankment 1, Saint Petersburg, 199034, Russia

**Keywords:** *Demodex folliculorum*, Infection, Eyelash, Skin, Age and gender structure, Contact lenses

## Abstract

Different morphotypes of *Demodex* from humans have been described. Among them, molecular studies have made it possible to discern between the *Demodex folliculorum and Demodex brevis*. Further studies showed two morphotypes of *D. folliculorum* harboured two different habits (human skin and human eyelashes), both of them with finger-shaped terminal opisthosoma difficult to differentiate and that can be assigned to *D. folliculorum,*

Thus, a complete morphometric study of the species, which in this study are referred to as *Demodex folliculorum* species complex, was carried out. From this morphometric and meristic study two distinct morphological forms (short and long) could be identified within the *Demodex folliculorum* species complex. These forms differ significantly in four out of the six biometric parameters we analysed: gnathosomal length and width, podosomal width, and opisthosomal length. Moreover, a comprehensive survey of the two morphotypes from different habitats (skin and eyelashes), was carried out in young people of Southern Spain. Therefore, an analysis of 104 asymptomatic students, which were contact lens wearers, and the presence of *D. folliculorum* was carried out. A statistical analysis based on Bayesian zero inflated Poisson GLM has been applied to our sample data. For the age group considered (18–24 years old), the overall *D. folliculorum* prevalence for skin face or eye infections (at least one of them) was 19.31 % (51 people), with a statistically significant higher prevalence in males men. Furthermore, there is a slight statistical correlation between the presence of *Demodex* in silicone hydrogel soft contact lens wearers*.* This study confirms: i) the existence of two morphotypes of *D. folliculorum* that appear segregated due to the parasitization microhabitats,ii) a higher prevalence of mites in men than in women, iii) the existence of a high number of statistically supported double infections (skin-eyelashes). It also provides epidemiological data on the prevalence of long and short forms of *D. folliculorum* in a healthy young population.

## 1 Introduction

Mites of the genus *Demodex*, show similar morphological characteristics due to the microhabitat where they develop: pilosebaceous follicles. *Demodex* species from humans are tiny mites (0.1–0.5 mm) with elongated appearance, four pairs of stumpy legs, and a long opisthosoma with annulations. This similar morphology could be due to convergent adaptative evolution among species of this genus (Thoemmes et al., 2014; Palopoli et al., 2015). After this permanent host association of *D. folliculorum,* studies on genome sequence and gene structure revealed an extensive genome reduction through relaxed selection and genetic drift, that coincided with an extreme reduction in the number of cells (Smith et al., 2022), representing an exceptional case among the Arthropods. This strategy could have determined the evolutionary history of *Demodex* species and the adaptation of different populations to specialized habitats.

*Demodex* species are the most common ectoparasites of humans and currently two species have been described: *Demodex folliculorum* (Simon, 1842) and *Demodex brevis* (Akbulatova, 1963) (Palopoli et al., 2015; Palopoli et al., 2014). Jing et al. (2005)) redescribed by SEM *D. brevis* based on the absence of spurs on each leg, only five pairs of palpal claws at the end segment of palpals, spindle hypostoma, and cone-like terminus. The former species are preferably located in the follicular infundibulum of the face (nose, cheeks, forehead, temples, and chin are favoured locations) and in eyelash follicles and prove to be the most prevalent. In contrast, *D. brevis* is settled in deeper sebaceous and Meibomian glands and the prevalence is much lower and recorded rarely (Zhao et al., 2012; Litwin et al., 2017). Phylogenetic analysis of 18S rDNA (Thoemmes et al., 2014; Zhao et al., 2012), as well as mitochondrial 16S rDNA sequences (Zhao and Wu, 2012; Zhao et al., 2013a), reveals the existence of two clearly differentiated species that at the intraspecific level, are geographically structured.

**Fig. 1:**
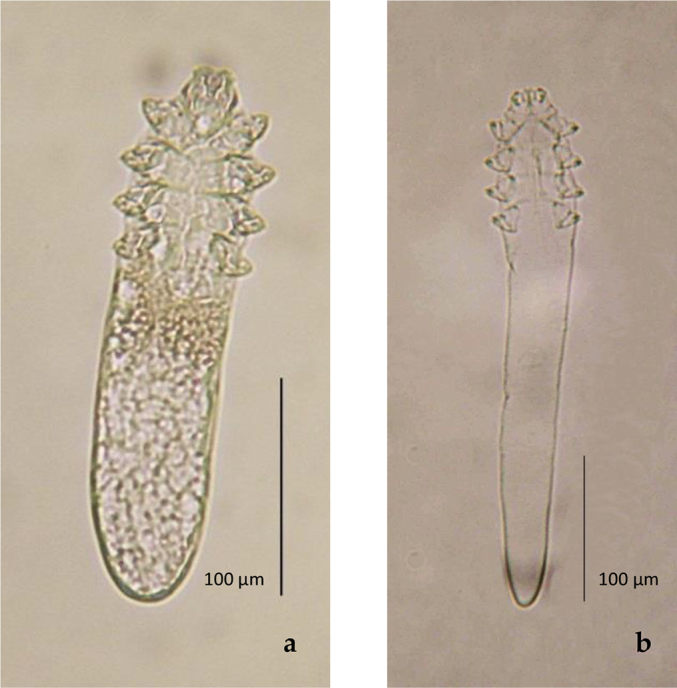
(a) *D. folliculorum* short bodied with finger-like terminus. Ventral female. Isolated from human skin. (b) *D. folliculorum* long-bodied with finger-like terminus. Ventral female. Isolated from human eyelashes.

Furthermore, Zhao et al. (2013b), observed four morphotypes (excluding males and immature forms): long and short-bodied *Demodex folliculorum* with finger-like terminus and *Demodex brevis* with finger- or cone-like terminus. The results of sequence analysis of 16S rRNA gene demonstrated that the genetic distances, among the three phenotypes with finger-like terminus indicated an intraspecific variation (Fig. 1) (Zhao et al., 2013a; Zhao et al., 2013b; Hu et al., 2014). However, the genetic distance among the three phenotypes with finger-like terminus and the one with cone-like terminus suggested an interspecific variation. Thus, in this study the phenotypes with finger-like terminus are referred to as *Demodex folliculorum* species complex. It is therefore worth considering that the shape of the opisthosomal terminus is a major morphological feature to differentiate these two species.

Numerous epidemiological surveys to determine the prevalence of *D. folliculorum* and *D. brevis* have been carried out in healthy people. Although the prevalence increases with the age of the host (Andrews, 1982; Liu et al., 2010), appears that most studies falling in the range of 10–20 % (Correa Fontt et al., 2020; Sevgen and Mor, 2019; Karaman et al., 2014; Cengiz et al., 2014; Cui and Wang, 2012; Vargas-Arzola et al., 2012; Yazar et al., 2008). These authors always isolated mites from the same microhabitat and they did not consider the relationships between populations from different locations (skin or eyelashes).

In addition, mite identification is done solely based on biometric differences and therefore *D. brevis* could be misidentified as the short type of *D. folliculorum* as can be seen, for instance, in the photograph showed in Tilki et al. (2017)). Moreover, the pathogenic role of *Demodex* species in humans has been demonstrated in several studies on the association between both populations *D. folliculorum* and ophthalmological or dermatological diseases that should be treated individually.

Among ocular pathologies, it is worth highlighting blepharitis (Liu et al., 2010; Kemal et al., 2005; Czepita et al., 2007; Zhang et al., 2018; Aumond and Bitton, 2020) along with chalazion (Liang et al., 2014; Yam et al., 2014), and corneal disease (Kheirkhah et al., 2007). Most authors in the past five years report that *Demodex* blepharitis accounts for ≥60 % of those with blepharitis (Rhee et al., 2023). Concerning the dermatological pathologies attributed to the presence of *Demodex*, rosacea stands out in order of importance (Bonnar et al., 1993; Moravvej et al., 2007; Türkmen and Türkoğlu, 2019; Sarac et al., 2020; Yazısız et al., 2019; Kubanov et al., 2019; Forton, 2022), followed by perioral dermatitis (Searle et al., 2021). Sebaceous hyperplasia with oily or mixed skin seems to favor *Demodex* proliferation (Zhao et al., 2011).

Thus, the objective of this work is, firstly, to know the prevalence values of *D. folicullorum* in healthy population of university students in southern Spain and to establish a relationship between the presence of the long and short finger-like terminus populations forms. Secondly, taking advantage of the exhaustive survey which incorporates contact lens wearers, the relationships between the presence of *D. folliculorum* and the use of contact lens will be analysed.

## 2 Materials and methods

In order to carry out the epidemiological study, the population was selected according to the following parameters: The exclusion criterion for people involved in this study was not to experience ocular or dermatological symptoms, not to be under corticosteroids or antibiotics therapy and an age not comprised between 18 and 24 years old.

The studied population consisted of 264 young people 186 women and 78 men (Table 1). Mean age of the study group was 20.77 years (age range from 18 to 24 years old).

**Table 1:** Descriptive statistics of biometric variables by origin. From left to right: Variable name, Mean, standard deviation (SD), interquartile range (IQR), Range, Asymmetry (Skewness) and the tailedness of the distribution (Kurtosis). In plain text, data from skin population (*N* = 28). In bold, data from eyelash population (*N* = 29).

Variable	Mean	SD	IQR	Range	Skewness	Kurtosis
Gn_L	23.96	1.91	3.55	[20.50–27.30]	0.17	−1.16
	**21.26**	**1.90**	**2.77**	**[18.20–24.60]**	**0.04**	**−0.97**
Gn_W	29.91	1.45	2.25	[27.30–32.50]	0.05	−0.75
	**25.87**	**2.18**	**3.18**	**[22.80–31.20]**	**0.74**	**0.21**
Pd_L	87.88	12.79	20.34	[50.25–103.53]	−1.00	1.02
	**80.90**	**11.92**	**19.85**	**[62.24–114.30]**	**0.49**	**0.57**
Pd_W	64.69	7.09	13.70	[55.60–76.20]	0.33	−1.37
	**58.20**	**3.87**	**4.45**	**[47.90–65.20]**	**−0.57**	**0.84**
Op_L	151.38	17.01	19.75	[104.19–183.30]	−0.87	1.26
	**254.92**	**31.90**	**55.12**	**[206.50–331.50]**	**0.33**	**−0.56**
Op_W	55.98	6.26	9.27	[44.90–64.90]	−0.50	−1.00
	**55.90**	**3.34**	**5.95**	**[51.00–63.80]**	**0.38**	**−0.74**
Tot_L	263.23	23.88	23.51	[204.20–295.30]	−1.31	1.50
	**357.09**	**39.07**	**61.22**	**[293.00–442.00]**	**0.19**	**−0.65**
Op_L/Tot_L	0.58	0.71	0.84	[0.51–0.62]	0.66	0.84
	**0.71**	**0.82**	**0.90**	**[0.70–0.75]**	**1.74**	**0.86**

On the other hand, in the study population, 104 students were users of soft silicone hydrogel soft contact lenses (75 women and 29 men).

## 3 Sample methods

Methods used to collect *Demodex* mites from humans included the cellophane tape method (placing adhesive tape on the face to stick to the mites on forehead and cheeks) (Li and Wang, 1986) and collection of eyelashes (Kemal et al., 2005). In this case we take three pieces of adhesive tape (3 cm × 1 cm) and plucking eyelashes (3 eyelashes from each eyelid), for determining parasitism of skin face and eyelashes populations respectively.

In accordance with the Helsinki II Declaration, before the start of the survey, all participants signed an informed consent, according to the model of Health Service of Andalusia (SAS).

### 3.1 Morphometric study

From the set of collected mites from skin and eyelashes, 28 individuals from the skin (short body type with finger-like terminus) and 29 specimens from the eyelashes (long body type with finger-like terminus) of *Demodex folliculorum* adult females were selected for morphometric study of both populations. The specimens were cleared in lactic acid and mounted in Hoyer's medium and carefully identified based on their morphological features (Jing et al., 2005). *Demodex brevis* was not detected.

Following previous investigations [40, 5, 9] we compared seven biometric variables of individuals from different origins (skin or eyelashes): Gnathosomal length (Gn_L); gnathosomal width (Gn_W); podosomal length (Pd_L); podosomal width, (Pd_W); opisthosomal length (Op_L); opisthosomal width (Op_W); total length (Tot_L); ratio opisthosomal length/total length (Op_L/Tot_L).

### 3.2 Statistical methods applied to morphometric study

Statistical methods applied to morphometrical and meristic study consisted in a descriptive study of the six biometric variables of individuals according to its origin. To do this, we made a summary table with the typical descriptive of measurements of the variables separated by groups, as well as graphically using Boxplots. We compare the mean lengths and widths of the measurements according to its origin using Barplots. With these types of graphs, we compare mean values of these variables, incorporating in turn a measure of dispersion such as the standard deviation.

In order to finding for significant differences in the morphological features (Gn_L, Gn_W, Pd_L, Pd_W, Op_L and Op_W) of the *Demodex* between the two populations (skin and eyelash), we decided to use a Hotelling's T2 test, a widely multivariate test used to deal with this kind of comparisons. Hotelling's T2 test procedures is equivalent to a MANOVA which uses the Hotelling-Lawley trace in its estimation. This type of MANOVA accounts for non-independence of the response variables. With this analysis, we check whether there exist significant differences globally between the morphological features of the two populations of the *Demodex*. Once the global differences between morphological features have been found, a Games-Howell post-hoc multiple comparisons procedure were carried out to detect among which morphological features these differences occur. Games-Howell is the more applicable test for pairwise comparisons when variances were nonhomogeneous, as in our case.

Hotelling's T2 test was fitted using *HotellingsT2Test* function of the ‘DescTools’ package (Vehtari et al., 2021), and its Games-Howell post-hoc multiple comparisons were conducted using *games_howell_test* function of the ‘rstatix’ package (Kassambara, 2024), via R software (R Core Team, 2021).

We use a support vector machine (SVM) as a predictive method for differentiating morphotypes of *Demodex* through the biometric analysis of their anatomy using values of morphological parameters. The purpose of a SVM is to construct optimal decision boundaries among classes in a decision plane. A decision plane separates a set of objects having different class memberships. To achieve this goal, we used *plot3d* and *rgl.planes* functions of the R packages ‘e1071’ (Meyer et al., 2023) and ‘rgl’ (Murdoch et al., 2022).

## 4 Statistical methods applied to epidemiological study

With the aim of determining how infection type and use of contact lenses affect to the number of *Demodex*, we construct a regression model. Generalized linear model (GLM) was used to conduct this regression analysis. Specifically, a zero-inflated Poisson GLM is used to model count data that has an excess of zero counts. Further, theory suggests that a separate process from the count values generates the excess zeros and that the excess zeros can be modelled independently. Zero-inflated models are used when the zeros observations have two different origins, these are, the “sampling zeros” and the “structural zeros”. The sampling zeros are assumed that were occurred by chance, that is, the subjects who are exposed to the outcome but may or may not register the event of interest during the study period. The structural zeros occurred when the subjects always produce zero counts. Therefore, a Bayesian zero-inflated Poisson GLM was fitted to determine the relationship among the total number of *Demodex* as dependent variable (counting), considering the infection type (categorized as 0 = no infection, 1 = eyelashes infection, 2 = skin infection, 3 = mixed infection (eyelashes and skin) and contact lenses (categorized as a dichotomous variable) as explanatory variables. Obviously, the explanatory variables were defined as fixed effects.

Bayesian zero-inflated Poisson GLM was fitted using “brms” package (Bürkner, 2017) via R software (Ripley, 2001). The function brm performs a Markov Chain Monte Carlo (MCMC) estimation to approximate the posterior distributions of the model parameters. Four chains with 5000 iterations each (warmup = 2500), for the first simulations the treedepth was set to 20, and default priors of the “brms” package were used. The R^2 value was also estimated with bayes_R2 function of “brms” package. It is obviously that we are not going to find multicollinearity problems in the model, however, we check the multicollinearity via variation inflation factors (VIFs) test using the check_collinearity function from the “performance” package in R (Lüdecke et al., 2021). Multicollinearity in the explanatory variables of the regression model is insignificant when VIF values were < 5 (James et al., 2013). Potential scale reduction factor (Rhat) for the stationary of the posterior distribution was reached when Rhat <1.01 (Vehtari et al., 2021). The significance of the parameters was hold whether their 95 % credible interval of the posterior distribution of the model parameters did not overlap zero. We plotted the uncertainty intervals - high-density intervals for the effects using the plot model function in the “sjPlot” package (Lüdecke et al., 2021).

Goodness of fit test was accomplished using “DHARMa” package (Hartig, 2024) which implements a method to standardize residuals for GLM (Dunn and Smyth, 1996; Gelman and Hill, 2007). This method based on the residuals allow for visual analysis just like residuals of linear models that would display standardized residuals uniformly distributed. We also implemented a Bayesian goodness of fit test to validate the candidate model using pp_check function from “brms” package. This method compares our observed data with the simulated data from the posterior predictive distribution. The R code to performance the statistical analysis is in Supplementary Material.

Fisher's exact test was applied in order to evaluate the associations between sex (man and woman) and the presence or absence of the mite infection (non-infected and infected). We used the fisher.test function from the package “stats” in R to carry out this test. To do this, we decided to performance Fisher's exact test separately in three different categories of infection, one for cases of only infection was detected in the eyelashes, and another for cases of only infection was detected in the skin and for both cases of infection (eyelashes and skin). The strength of the associations was checked via Cramer's V test using assocstats function of the “vcd” package (Meyer et al., 2023). When the values of Cramer's V test (ES) are equal or less than 0.2 (ES ≤ 0.2), the association is considered as weak, if 0.2 < ES ≤ 0.6, the association is moderate, and if ES > 0.6 the associations is strong.

## 5 Results

### 5.1 Morphometric study

Adult females of *D. folliculorum* and *D. brevis* can be separated by morphometrical features as: total length, the position of the vulva at the level of the coxae IV in *D. folliculorum* and behind them in *D. brevis*. Moreover, *D. folliculorum* presents seven pairs of palpal claws and shows tiny spurs on each leg while in *D. brevis* only five pairs of palpal can be located and spurs are not showed on their legs. Furthermore, *D. brevis* shows a pointed shape of the end of the opisthosoma, while in *D. folicullorum* is rounded.

In this study, no morphological features were detected between *D. folliculorum* species complex. Thus, a complete biometric study was carried out in specimens from skin and eyelashes.

The total length range of the individuals analysed varies between those coming from the skin (204.20 to 295.30 μm), versus those coming from the eyelashes (293.00 to 442.00 μm). The mean in the total length is greater in the case of the eyelashes (average: 263.23 μm, SD: 23.88) compared to the skin (average: 357.09 μm, SD: 39.07) (Table 1).

**Fig. 2:**
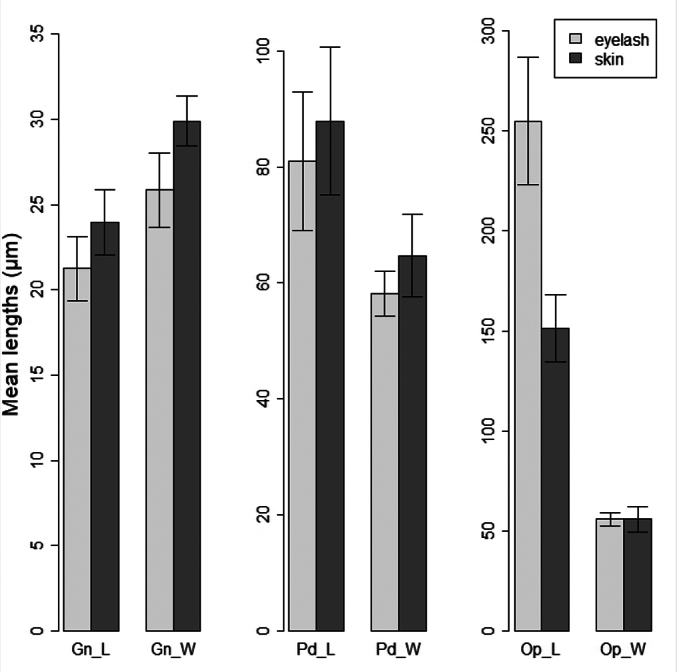
Barplots showing the means values with its standard deviations for the six variables considered.

Means values and its standard deviations for the six variables considered are represented in Fig. 2.

**Fig. 3:**
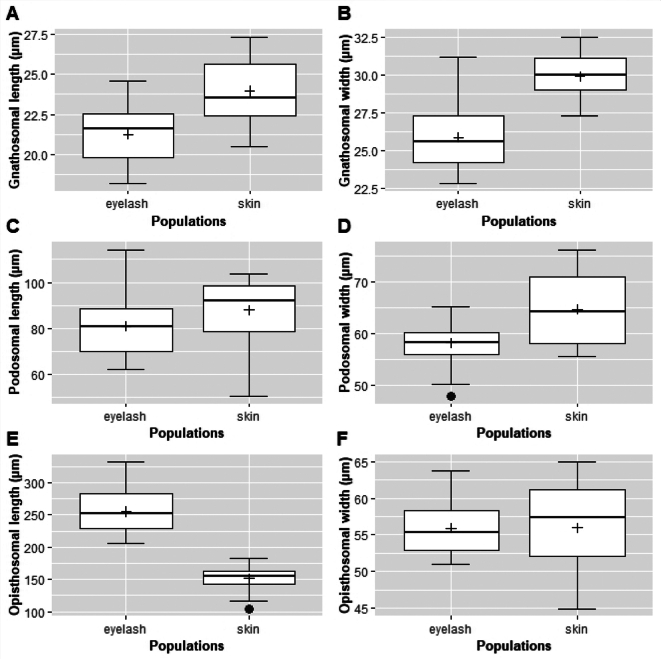
Boxplots represent the values of the six variables according to origin (skin and eyelash): The quartiles of each distribution are represented by the top and bottom of the boxes, the median is the line across the box, the mean value is represented by the cross inside the box. Upper and lower limits are represented by the vertical solid lines.

The statistics relied with the distribution of both populations are showed in Fig. 3. A Hotelling's T2 test was performed to determine the effect of population (skin and eyelash) on morphological features (Gn_L, Gn_W, Pd_L, Pd_W, Op_L and Op_W). The Hotelling's T2 test was detected globally significant difference among the types of population on morphological variables (Hotelling's T2 = 73.648, *p*-value <2.2e-16). According to Table 2, there exists significance differences in the completely pairwise comparisons but Op_W variable, with a p-value = 0.953.

**Table 2:** Pairwise comparisons between populations using Games-Howell post hoc test.

**Variables**	**Comparisons**	**Estimate**	**Low CI 95 %**	**Upper CI 95 %**	**p-adj**
Gn_L	Eyelash-skin	2.70	1.69	3.72	1.77e-6
Gn_W	Eyelash-skin	4.04	3.05	5.02	7.87e-11
Pd_L	Eyelash-skin	6.98	0.41	13.5	0.038
Pd_W	Eyelash-skin	6.49	3.42	9.56	1.11e-4
Op_L	Eyelash-skin	−104	−117	−89.9	1.11e-12
Op_W	Eyelash-skin	0.08	−2.62	2.78	0.953

Six biometric patterns as predictor values were used to construct support vectors in the first phase. First training phase and SVM results for two biometric patterns dependent variable to be classified: *Demodex* morphotypes (skin or eyelashes). Correlation matrix shows high correlation of Gn_W with Gn_L and Po_L features, and that determine that in the second phase of the study Gn_W were excluded of analysis. In order of importance, we selected as predictors the following variables: Gn_L, Pd_W and Op_L (Table 2).

**Fig. 4:**
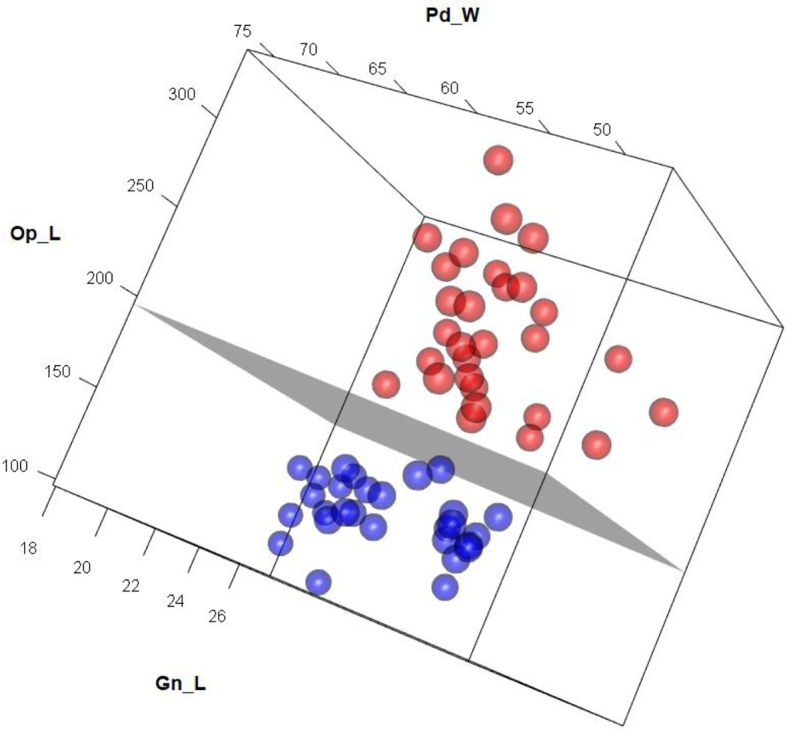
Three-dimensional feature space with the two Demodex classes defined by variables Gnathosomal length (Gn_L), Podosomal width (Pd_W and Opistosomal length (Op_L). Red dots indicate eyelash samples, while blue dots represent skin mites. The boundary decision hyperplane is shown in grey. The scales are in μm. (For interpretation of the references to colour in this figure legend, the reader is referred to the web version of this article.)

*Demodex* from skin (blue) and from eyelashes (red) have been represented in a point cloud (3D scatterplot) using the support vector machine technique. We have found a perfect classification of both samples through the maximum margin hyperplane (Fig. 4).

The corresponding classifier is Clas = −14.9201 + 0.009214008*Gn_L + 0.015592936* Pd_W -0.082217298 * Op_L. When the value of Clas >0, the concerned *Demodex* mites comes from the eyelashes, while Clas <0 corresponds to a skin *Demodex* (Fig. 3).

### 5.2 Epidemiological study

With regard to the epidemiological data on demodectic infection, the following can be highlighted.

The overall *D. folliculorum* prevalence for skin face or eye infections (at least one of them) was 19.31 % (51 people) (Table 1). When different data groups were considered, prevalence for skin face was 15.53 % (365 specimens isolated) and 16,28 % for eyelashes (153 specimens isolated). From these two populations of *Demodex* those specimens in better conditions were chosen for morphometrical studies (28 and 30 respectively).

The mean of *D. folliculorum* count for each population was 0.31 mites per eyelash and 0.96 mites per cm^2^ respectively (infection index was calculated as in Rivera et al., 2013 (Rivera et al., 2013)).

**Fig. 5:**
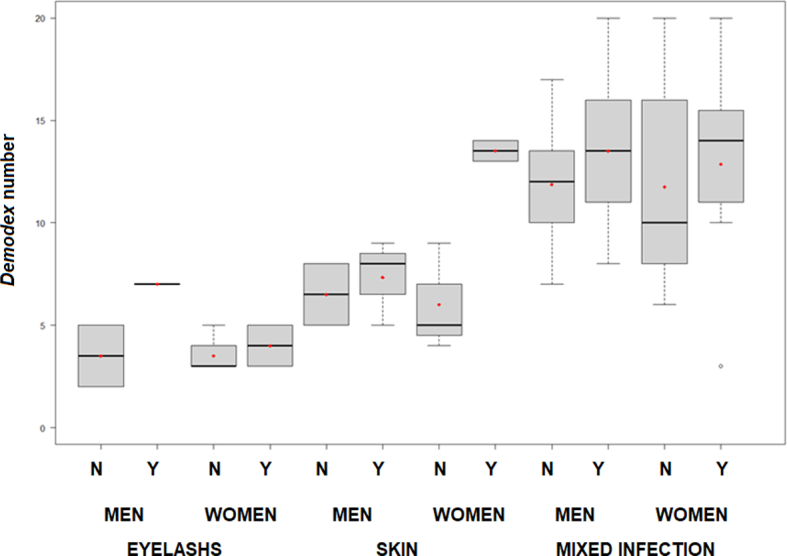
Boxplot of men (M) and woman (W), with *Demodex folliculorum* species complex positive infection (eyelashes, skin or mixed infection), considering the use of contact lenses (Y = yes, N = no). Red points represent the median value. (For interpretation of the references to colour in this figure legend, the reader is referred to the web version of this article.)

When sex distribution was analysed, for each population, 7.19 % of infected people were men and 8.33 % women if *D. folliculorum* eyelashes population were considered. Similar results show the infection of skin population (7.95 % for men and 8.33 % for women) (Table 3). The effect of contact lens use determined higher percentages of infection in both men and women (Table 4, Fig. 5), going from an infection that affects 19.6 % of non-user men to 63.5 % of users. In the case of women, something similar occurs, such that the percentage of infection ranges from 10.7 % to 23.9 %.

**Table 3:** Prevalence values considering all people surveyed. Total number of mites collected and Infection Index (as number of mites per lash or number of mites per cm^2^, depending on the sampling site considered).

All Volunteers Considered		*D. folliculorum* from eyelashes	*D. folliculorum* from skin	Mixed infection
Men (78)	No. Infected people	19	21	17
	Prevalence (%)	24.36	26.92	21.79
	No. mites	77	173	–
	Infection Index	0.02	0.07	–
Women (186)	No. Infected people	22	22	15
	Prevalence (%)	11.83	11.83	8.10
	No. mites	76	192	–
	Infection Index	0.02	0.08	–
Total (264)	No. Infected people	41	43	32
	Prevalence (%)	15.53	16.30	12.12
	No. mites	153	365	–
	Infection Index	0.05	0.15	–

**Table 4:** Prevalence values considering the use of contact lenses: number of cases (in bold) and percentage of prevalence (in brackets).

	Use of contact lens	No (%)	Yes: (%)	Total: (%)
Men	N	**56** (71.79)	**22** (28.20)	**78** (100)
	Only eye inf.	**2** (3.57)	**1** (4.54)	**3** (3.84)
	Only skin inf.	**2** (3.57)	**3** (13.63)	**5** (6.41)
	Mixed inf.	**7** (12.50)	**10** (45.45)	**17** (21.79)
	Total inf.	**11** (19.64)	**14** (63.63)	**25** (32.05)
Women	N	**140** (75.26)	**46** (24.73)	**186** (100)
	Only eye inf.	**4** (2.85)	**2** (4.34)	**6** (3.22)
	Only skin inf.	**3** (2.14)	**2** (4.34)	**5** (2.68)
	Mixed inf.	**8** (5.71)	**7** (15.21)	**15** (8.06)
	Total inf.	**15** (10.71)	**11** (23.91)	**26** (13.97)

Fisher's exact test for associations between sex (man and woman) and the existence of demodicosis (uninfected versus infected) for both cases of infection (eyelashes and skin) show a p-value = 0.0011, indicating significant differences due to sex. The value obtained for Cramer's V (0.21) indicates a moderately significant strength of association between *Demodex* infection and the sex of the individual.

If we consider exclusively the infection in the eyelashes (Table 2, Fig. 2), we obtain a value of *p* = 0.0049 in the Fisher test, which indicates a significant difference when considering sex. In this case, the Cramer's V value is 0.18, indicating a weak significant strength of association. When we consider skin infection, the value obtained for the Fisher test is 0.0008, which indicates significant differences according the sex, although in this case the strength of association is moderately significant (Cramer's V of 0.22). Therefore, we can say that the proportion of infected is not the same depending on whether the potential host is a man or a woman, assuming a significance level of 5 %.

With regard to the number of specimens of *Demodex folliculorum*, ranged from 1 to 8 mites in eyes whereas the number of mites from skin ranged from 2 to 16, being the most infected 6 people with 12 mites. Concerning mixed infections, 32 people showed infections in skin face and eyelashes simultaneously (Table 1).

**Fig. 6:**
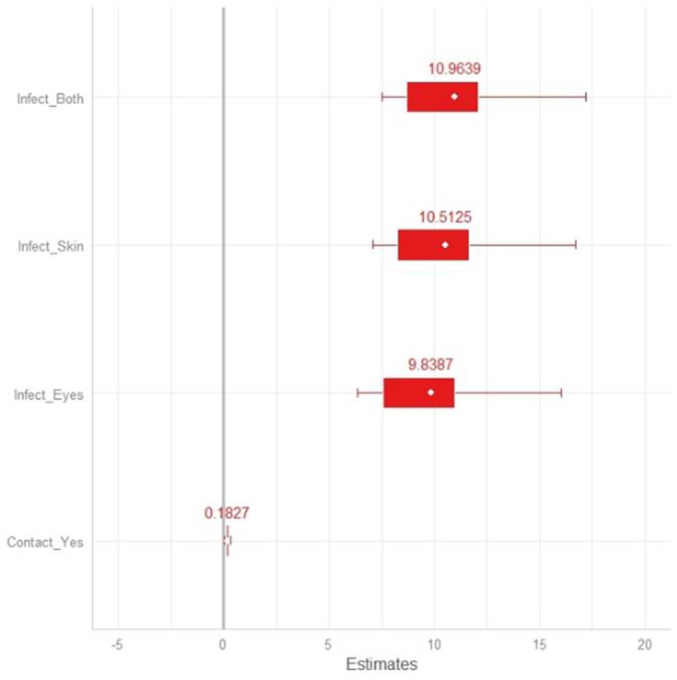
Influence of factors on the number of *Demodex folliculorum* species complex in young people. Estimated coefficients (and 95 % high-density intervals) of the covariates for the explanatory variables. 95 % high-density intervals overlapping 0 (solid vertical line) indicate a non-significant coefficient. No infection and no contact lenses are the baseline categories for both categorical explicative variables.

Table 5 and Fig. 6 show the results of the Bayesian zero-inflated Poisson GLM fitted. Zero-inflated model split the process in two parts; the first is a summary of the full model and can be interpreted as a standard Poisson model. The second part (the zero component) predicts whether or not the outcome is a certain zero (the logistic part of the model). A common way of interpreting logistic regression models is to exponentiate the coefficients, which places the coefficients in an odds-ratio scale. With zero-inflated models, the logistic part of the model predicts non-occurrence of the outcome. In this regard, the zi-intercept of the model can be interpreted as follows: the baseline odds ratio of being among the never infected is exp. (0.02) = 1.02.

**Table 5:** Parameter estimates for the Bayesian zero-inflated Poisson GLM. From left to right: Predictor variable, estimated coefficient, estimate error and the lower and upper credible 95 % intervals (CI). Non-overlapping CIs with zero imply a significant (*p*-value <0.05) difference between groups. ZI-intercept refers to the intercept of the zero-inflated model part. No infection and no contact lenses are the baseline categories for both categorical explicative variables.

Predictor	Estimate	Est. Error.	lower-95 % CI	upper-95 % CI
Intercept	−8.54	3.65	−17.87	−4.71
ZI-intercept	0.02	0.02	0.00	0.07
Infect_Eyelashes	9.84	3.66	6.01	19.14
Infect_Skin	10.51	3.65	6.69	19.82
Infect_Both	10.96	3.65	7.15	20.28
Use of contact lens_Yes	0.18	0.09	0.01	0.36

We found significant effects in all the categories for the two response variables considered in the model. People with infection in eyes, skin and both concerning people without any infection stage, increased significantly the total abundance of *Demodex* with coefficients and credible intervals ranged at values: 9.84 (6.01, 19.14), 10.51 (6.69, 19.82) and 10,96 (7.15, 20.28) respectively, (see Table 5 and Fig. 6).

It is noteworthy that the average age of contact lens users in Spain is around 30 years, a figure almost equal to the average age of contact lens users worldwide (31 years). The overall percentage of silicone hydrogel soft contact lens fittings is 37 %. Women constitute the majority of contact lens users in Spain, representing around 59 %, a value slightly lower than that of the rest of the world (67 %) (Santodomingo et al., 2012).

According to the model estimate, people who wear contact lenses significantly increased the total abundance of *Demodex* in relation to those who do not wear them, with a statistically significant parameter: 0.18 (0.01, 0.36) but with a minimal influence in the development of ocular infection (Fig. 6). Bayesian R^2^ for zero-inflated Poisson GLM model explained 87.42 % of the variance of the number of *Demodex*, and VIFs values were all <3 for the explanatory variables in the model.

The graphical goodness-of-fit of the model according to the “DHARMa” package and using the pp_check function of the “brms” package, can be found in the Supplementary Material, along with the R code for the entire statistical analysis.

## 6 Discussion

### 6.1 Morphometrical aspects

The molecular identification of four phenotypes of human *Demodex* mites demonstrated long- and short-bodied D. folliculorum with finger-like terminus and *D. brevis* with finger or cone-like terminus (Zhao et al., 2013a). The molecular data permit to reclassification *D. brevis* with finger-like terminus, formerly classified as *D. brevis*, to be *D. folliculorum* and it might be a morphological variant of *D. folliculorum* classical form.

We found significant differences in four of six parameters considered with the exception of podosomal length (Pd_L) and opisthosomal width (Op_W) (Table 2). The SVM allows delimiting the maximum margin hyperplane that provides a perfect classification of both populations (eyelash and skin) (Fig. 4). In the reviewed literature, epidemiological papers have only considered one of both habitats and have not been reported correlations between both populations.

*Demodex* species from humans show a reduced transfer capacity among host and low dispersal rates (Palopoli et al., 2015). In this context it may be possible to hypotetized the segregation of two morphotypes in *D. folicullorum* as response to an adaptation on characteristics of two specialized microhabitats (hair follicles and sebaceous glands) that determine the difference registered in the size of each mite morphotype.

Based on the morphometric results obtained in this work, and the statistical analysis applied, we can conclude that the two morphotypes of *D. folliculorum*, long and short body with finger-like terminal isolated from different habitats (eyelashes and skin), can be differentiated by applying a simple equation.

### 6.2 Epidemiological aspects

In the reviewed literature, epidemiological papers have only considered one of both habitats and have not been reported correlations between both populations. In addition, there have been no morphological and/or biometric features or references for identification of mite specimens in each paper. There are even some studies where *D. brevis* is determined based on scarce biometric criteria, exclusively, which may lead to misidentifications (Tilki et al., 2017).

It is well reported that the prevalence values of *Demodex* species increase as age host increases, affecting more than 80 % of those older than 60 years and 100 % of those older than 70 years (Cheng et al., 2015). Studies based on university populations determined a *Demodex folliculorum* prevalence ranging between 2 % and 27 % (Correa Fontt et al., 2020; Murphy et al., 2020; Vargas-Arzola et al., 2020). Generally, clinical blepharitis is associated with high rates of *D. folliculorum* infestation (prevalences ranging from 29 % to 90 %) (Zhang et al., 2020).

Thus, in this study, we selected a homogenous age group (from 18 to 23 years old) and the prevalence in this group for each *Demodex* population varies slightly from those obtained in other studies and agrees with the results of different authors for the same habitats.

So, with regard to *Demodex folliculorum* from skin, prevalence values ranged between 7 % and 34 % (Litwin et al., 2017; Tilki et al., 2017; Bonnar et al., 1993; Godínez-Hana et al., 2004; Lazaridou et al., 2010; Sattler et al., 2012; Murillo et al., 2014) This variability is likely due to different age groups considered. When data obtained in this study could be compared with the same age group, the value was lower than other studies and more similar to 17 % obtained by Lazaridou et al. (Lazaridou et al., 2010).

The prevalence values for studies concern with *Demodex* from eyelashes ranged between 8 % and 19 % (Kamoun et al., 1999; De Freitas Yamashita et al., 2011; Sędzikowska et al., 2018). The value we obtained (15.90 %) agrees with most of the studies in the world for this age group.

However, in the reviewed literature, only two studies considered isolated independent mite populations of eyelashes and skin and they did not establish the possibility of cross-linkages between symptom aetiologies due to only one of their populations (Aumond and Bitton, 2020; Jaime-Villalonga et al., 2018).

Moreover, it was found that a greater number of men than women were infected with *Demodex*. This may be because men's skin has oilier skin and a greater amount of sebum, which these mites feed on, making the microhabitat in men more suitable for them (Man et al., 2009). It has been reported that androgenic stimulation leads to an increase in size and secretory activity of sebaceous glands among men (Roh et al., 2006; Ailawadi, 2022), determining a higher sebum production and larger pore size, with average values for sebum secretion being significantly higher in men than in women over of 20 years (Jalbert and Rejab, 2015).

With regard to the infection index, as expected for healthy people according to Rivera el al. (Rivera et al., 2013), was 0.31, lower than the value of 0.5 mites per eyelash, minimum to consider the existence of overpopulation of mites. In the case of skin face, infection intensity was 0.96 mites/cm2, much lower than 5 mites/cm2 than some authors establish like significantly increase the risk of cutaneous demodicosis (Rivera et al., 2013; Sędzikowska et al., 2018).

Thus, concurrent infection of eyelashes and skin face is much higher (32 cases) than expected (6.52 cases). This is an important data because, firstly, implies that the taxonomic status of both populations must be resolved to discriminate if they are different species sharing similar microhabitats cause their analogous transmission mechanism or, by contrast, the same species that adopts different morphotypes to adapt to slightly different microbugs. This fact implies that the pathogenic role of the two different morphotypes of *D. folliculorum* in the hair follicle of the skin and lashes remains unclear because the presence of *Demodex* in skin face can influence ophthalmologic disease and vice versa like have been reported by Sędzikowska et al. (2018)).

Two forms of *D. folliculorum* females have been reported from healthy people of Southern Spain, formerly named long and short forms clearly differentiate in base of biometry of gnathosome length and width, podosome width and opisthosome length) (de Rojas et al., 2012) and genetic analysis (Zhao and Wu, 2012; Zhao et al., 2013a). In this paper, the authors signaled the existence of two morphotypes of females of *D. folliculorum* corresponding with the populations of the skin and the eyelashes and showed some molecular differences in COI sequences between them. Obviously, the correct diagnosis could help to establish if one of them affect mostly to eye pathologies and the other to dermatologic disease, or even one of these morphotypes can be involved in both pathologies.

In this study, the overall percentage of contact lens users is slightly higher (43.26 %) than the overall Spanish mean, possibly because the age group considered is the one with the highest number of contact lens wearers and the higher number of women included in this study.

After reviewing the literature, we found only one article that discusses the relationships between contact lens wearers and the presence of *Demodex* (Jalbert and Rejab, 2015). The study aimed to determine whether *Demodex* infestation is more common in contact lens wearers and to evaluate the effects of its presence on the ocular surface. The authors concluded that contact lens wearers have higher *Demodex* numbers than people do not use it.

However, it is noteworthy that there are several limitations to relating the results of this study to the present work. Firstly, the present work is limited to the young age group 18–22 years, which eliminates the bias of increasing prevalence with age. On the other hand, only healthy young people with no ocular or dermatological symptoms are involved. Finally, statistical methods are used that eliminate the bias of high masses with numerous negatives (zero-inflate).

According to our findings, there is a slight statistical correlation between the presence of *Demodex* and the use of contact lenses. This fact agrees with the line of argument by Jalbert and Rejab (Jalbert and Rejab, 2015) that suggested a higher number of *Demodex* in contact lens wearers.

Thus, wider further studies on the prevalence in healthy people of short and long round terminus types of *D. folliculorum* and the association of both mite populations with ophthalmological and dermatological disease, and comprehensive surveys in different groups of soft silicone hydrogel contact lens wearers must be carried out.

## 7 Conclusions

This study confirms:

i) the existence of two morphotypes of *D. folliculorum* in different microhabitats (skin and eyelashes);
ii) a higher prevalence of *D. folliculorum* in men than in women;
iii) a high number of statistically supported double infections (skin-eyelashes).

It also provides epidemiological data on the prevalence of long and short forms of *D. folliculorum* in healthy young population.

## Funding

This research was funded by VII Plan Propio de Investigación de la Universidad de Sevilla, Spain and Grant numbers: 1, POAI 2023-2024: BIO294_2023 Universidad de Jaén, Spain.

## Author Contributions

**Conceptualization**, M.D.R. and F.J.M.; **Sampling**: S. S-C and I.D. **Statistical analysis**, F.J.M, and A.L-M.; **Writing – original draft preparation**, M.D.R. and F.J.M.; **Writing – review and editing**, M.D.R., F.J.M., A.L-M, S. S-C.; **supervision**, F.J.M and M.D.R. All authors have read and agreed to the published version of the manuscript.

## Declaration of competing interest

The authors declare no conflict of interest.

## Appendix A Supplementary data

Supplementary material Image 1
