# Supplementary material for: Epidemiological survey of two morphotypes of Demodex folliculorum (Prostigmata: Demodicidade) in young people from southern Spain
Source: Parasite Epidemiol Control. 2024 Sep 28;27:e00381. doi: 10.1016/j.parepi.2024.e00381 (PMC11497486; doi:10.1016/j.parepi.2024.e00381)
Supplement: Supplementary file 1 — Supplementary material [file mmc1.pdf]

Supplementary Material I

Epidemiological survey of two morphotypes of Demodex folliculorum (Prostigmata: Demodicidade) in young people from southern Spain

Márquez, F. J.[a], López-Montoya, A. J.[b], Sánchez-Carrión, S.[c], Dimov, I.[d] De Rojas, M.[c]

[a]Department of Animal and Vegetal Biology and Ecology, Jaén University, Campus Las Lagunillas, s.n., E-6 23071, Jaén, Spain.

[b]Department of Statistics and Operational Research, Jaén University, Campus Las Lagunillas, s.n., E-6 23071, Jaén, Spain.

[c]Department of Microbiology and Parasitology, Faculty of Pharmacy, University of Sevilla, Profesor García González 2, 41012 Seville, Spain.

[d]Department of Human Anatomy, State Pediatric Medical University, Litovskaya St. 194100 St. Peters-burg, Russia.

Zero-inflated Bayesian GLM

The following R code shows the Bayesian zero-inflated Poisson GLM fitting according to brms package (Bürkner, 2017).

Bayesian zero-Inflated Poisson GLM code in R

```
library(ggplot2)
library(DHARMa)
library(brms)
library(broom)
library(loo)
library(bayesplot)
library(rstan)
library(performance)
library(sjPlot)

### Load de database ###
Dem_epid<-read.delim("C:/.../database.txt")
demodex<-as.data.frame(Dem_epid)


### categorical variables ###
demodex$Contac_l<-factor(demodex$Contac_l)
demodex$Infect_T<-factor(demodex$Infect_T)


##########################################################################
################# Bayesian zero-Inflated Poisson GLM model ###################
##########################################################################
```

```r
set.seed(123)

m1 <- brm(Dem_N ~ Infect_T + Contac_l, data = demodex,
               iter=5000,
               control = list(max_treedepth = 20),
               family="zero_inflated_poisson")
summary(m1)

#### Extracting the R-squared of the model ####
bayes_R2(m1)


#### Check the multicollinearity ####
check_collinearity(m1)


#### Plot the coefficients with their uncertainty intervals ####
theme_set(theme_sjplot())
win.graph()
p1<-plot_model(m1, bpe = "mean", bpe.style = "dot", type = "est", sort.est = TRUE,
               transform = NULL,vline.color = "gray", show.values = TRUE, value.offset = .2,
               title = "", digits = 4, axis.lim=c(-1,17), axis.title = c("Estimates"),
               axis.labels=c("Contact_Yes", "Infect_Eyes", "Infect_Skin", "Infect_Both"),
               dot.size=1.5)
p1


############# Checking the model with DHARMa package ###############

model.check1 <- createDHARMa(
  simulatedResponse = t(posterior_predict(m1)),
  observedResponse = demodex$Dem_N,
  fittedPredictedResponse = apply(t(posterior_epred(m1)), 1, mean),
  integerResponse = TRUE)

win.graph()
plot(model.check1)


#### Bayesian checking with brms package ####
win.graph()
pp_check(m1, nsamples = 1000) + xlim(0,25)
```

References

Bürkner, P.-C. 2017. Brms: An r package for bayesian multilevel models using stan. *Journal of Statistical Software* **80**: 1–28.

R Development Core Team. 2021. R: A Language and Environment for Statistical Computing. R Foundation for Statistical Computing. Vienna, Austria. Available at: https://www.R-project.org.

Supplementary Material II

Graphical goodness of fit test for Bayesian zero-inflated Poisson GLM via "DHARMa" package

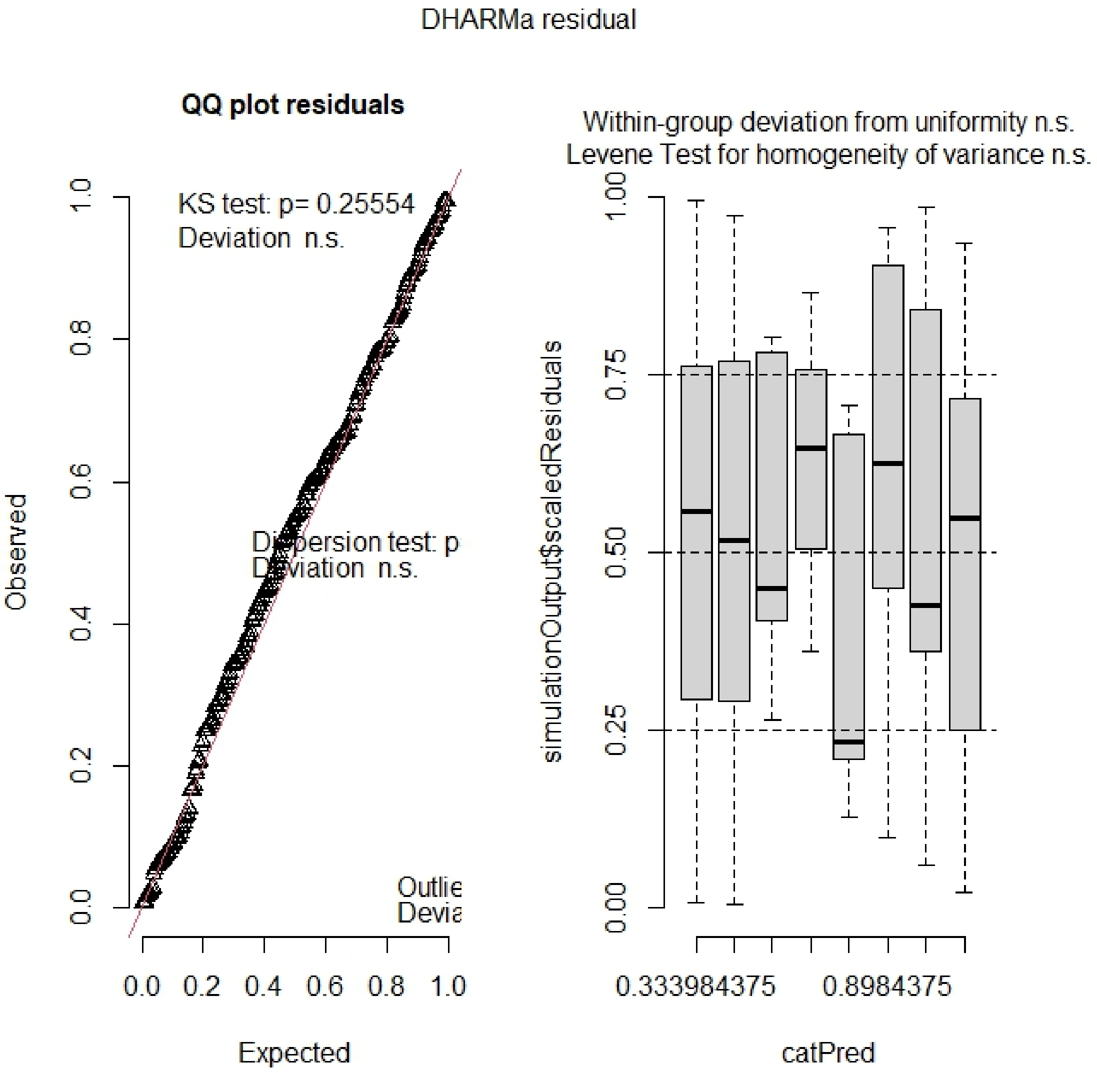

Bayesian goodness of fit test to validate the zero-inflated Poisson GLM model using pp_check function from “brms” package

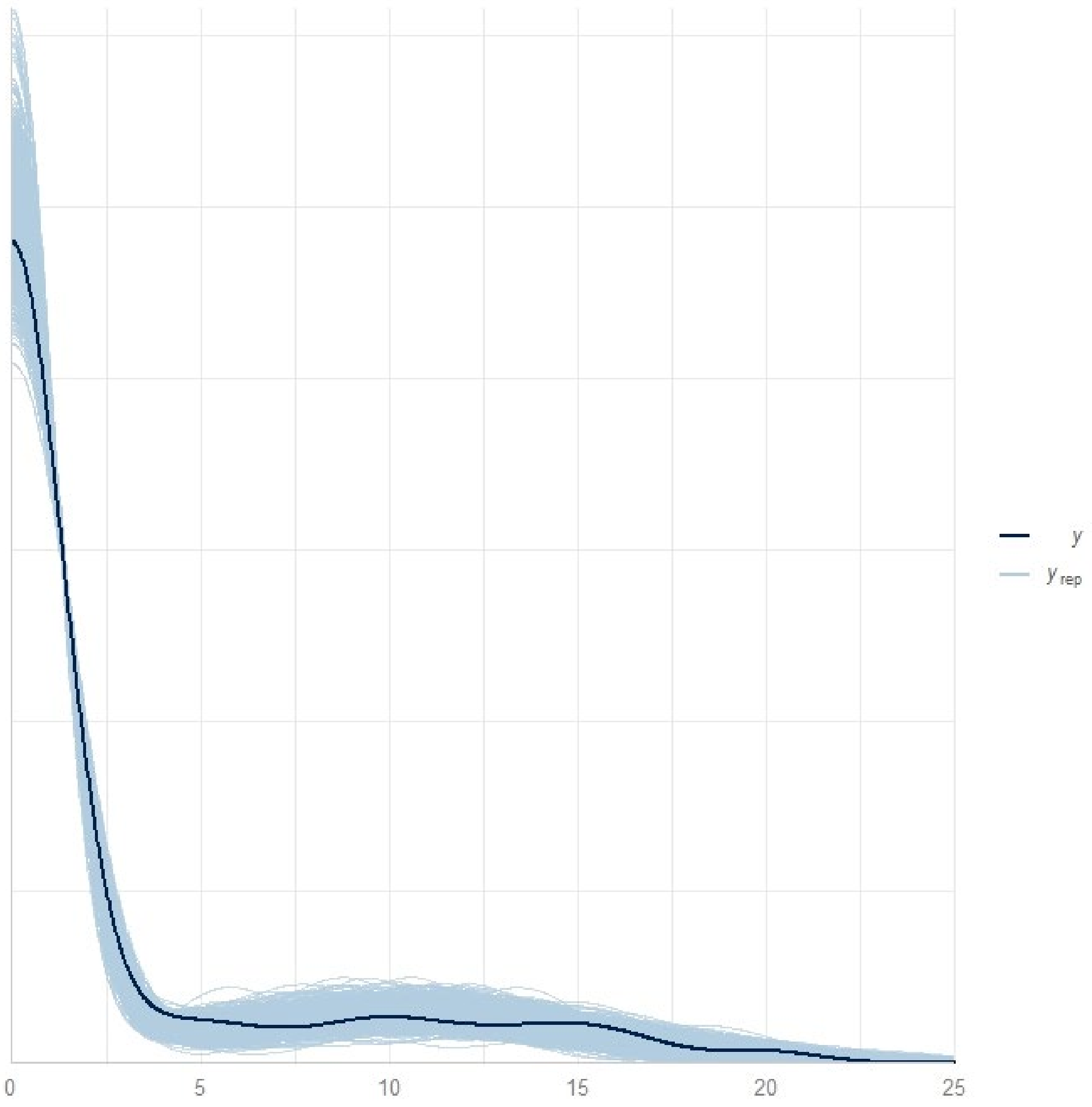

```
> #### Extracting the R-squared of the Bayesian zero-inflated Poisson GLM model ####
> bayes_R2(m1)
   Estimate      Est.Error     Q2.5       Q97.5
R2 0.8741544   0.01294294   0.8420376   0.8917112
>
```

```
> #### Check the multicollinearity ####
> check_collinearity(m1)
Low Correlation
 Term  VIF    VIF     95% CI        Increased SE   Tolerance   Tolerance 95% CI
 Infect_T     1.01   [1.00, 309.09]     1.01          0.99         [0.00, 1.00]
 Contac_l     1.01   [1.00, 309.09]     1.01          0.99         [0.00, 1.00]
>
```
